# Parental acceptability of silver diamine fluoride: The UK and US experiences

**DOI:** 10.1111/ipd.13195

**Published:** 2024-04-26

**Authors:** Laura Timms, Sooji Choi, Zoe Marshman, Helen Rodd, Anne R. Wilson, Tamanna Tiwari

**Affiliations:** ^1^ School of Clinical Dentistry University of Sheffield Sheffield UK; ^2^ Children's Hospital Colorado Aurora Colorado USA; ^3^ School of Dental Medicine University of Colorado Anschutz Medical Campus Aurora Colorado USA

**Keywords:** caries management, community paediatric dentistry, minimally invasive dentistry, restorative dentistry/dental materials

## Abstract

**Background:**

International data suggest that parents may have reservations about the use of silver diamine fluoride (SDF).

**Aim:**

The aims of this study were to: (1) examine the acceptance of parents/carers towards the use of SDF for the management of caries in children's primary teeth in secondary care dental settings in the UK and the United States and (2) determine which factors may affect the acceptance of the use of SDF.

**Design:**

This was a cross‐sectional questionnaire of SDF acceptability, completed by parents of young children. It was validated and adapted to local populations. Data were analysed with descriptive and inferential statistics.

**Results:**

Of the 113 Sheffield parents, 73% reported that they would accept SDF treatment of children's posterior teeth, with 58% reporting this for anterior teeth. Parents having less concern about posterior aesthetics had a statistically significant effect on reported acceptance of SDF (*p* = .013). In the Colorado sample (*n* = 104), 72% reported that they would accept SDF on posterior teeth, and 58% reported that they would accept SDF on anterior teeth. Concerns about aesthetics had an effect on decreasing SDF acceptance overall (*p* = .0065) in anterior (*p* = .023) and posterior teeth (*p* = .108).

**Conclusion:**

The majority of parents in the two study populations accepted the treatment using SDF. However, concern about aesthetics had an influence on acceptability.

**Why this paper is important to paediatric dentists**

- This study highlights the acceptability of SDF in different international populations, 58% for anterior teeth and 72%–73% for posterior teeth.
- This study provides evidence that parents may accept SDF as a treatment modality despite their unhappiness with the resultant aesthetics.

## 1 INTRODUCTION

Inequalities pervade the distribution of caries in both the UK and the United States, with those from areas of deprivation suffering disproportionately with both a higher prevalence and severity of caries. Recent national surveys in the UK have reported caries into dentine in 10.7% of 3‐year‐olds and 23.4% of 5‐year‐olds.^1^, ^2^ Furthermore, these lesions are often untreated, or managed with approaches that have poor outcomes.^1^, ^3^ Those from areas of deprivation are less likely to receive restoration of these carious teeth. In the United States, disparities in dental caries are also more prevalent in children of lower socio‐economic status and minority race/ethnic groups.^4^, ^5^ The 2021 Oral Health in America reported one in four preschool‐aged children have dental caries experience in their primary dentition.^6^ Other reports reflect the same trends: 23% of US children aged 2–5 years reported to have caries, and 10% of this age group having untreated caries.^7^

In view of this high caries prevalence, there is a clear need to explore alternative treatment approaches, such as silver diamine fluoride (SDF), for the management of young patients with early childhood caries. There is a strong and compelling evidence base for the effectiveness of minimally invasive caries management options.^8^ Silver diamine fluoride is one such minimally invasive approach which requires less cooperation than other surgical treatments. As such, it is an important treatment modality to offer for patients who cannot accept other evidence‐based approaches.

Silver diamine fluoride has been used for decades internationally and has proven efficacy in arresting caries progression in the primary dentition.^9^ Multiple systematic reviews demonstrate consistent evidence from randomised controlled trials with success rates for caries arrest between 65 and 91%.^9^ It was licensed for use in the UK in 2017 and the United States in 2014; both these approvals, however, were for the treatment of dentine hypersensitivity. In the UK, while not licensed for caries arrest, it is possible to use SDF off‐licence for this purpose where appropriate. The British Society of Paediatric Dentistry produced a standard operating procedure for SDF use in 2020.^10^ In 2016, the US Food and Drug Administration granted SDF breakthrough therapy status in relation to its use for caries arrest in young children, for which full approvals were sought and recently granted.^11^ The American Academy of Pediatric Dentistry introduced guidelines in 2017 for the use of SDF related to caries arrest.^12^

Following the application of SDF, carious tooth tissue becomes black, which has been highlighted by the international paediatric dentistry community as a perceived barrier to its implementation.^13^, ^14^, ^15^, ^16^ A systematic review demonstrated varied acceptability of SDF by parents with rates of 0%–100% cited across different studies.^17^ There were different methodologies used within these studies; nonetheless, the range of results does indicate a disparity in the degree of acceptability across different locations. Data from the United States (New York) suggest that parental acceptability rates are likely to be greater when SDF is applied to posterior teeth than applied to anterior teeth, and when the alternatives to SDF involve pharmacological interventions.^18^ To date, there is a paucity of comparable data about parental perspectives regarding SDF use in the UK. Nevertheless, as little is known about parental acceptability of this approach, particularly in different countries and service settings, this presents an important area of initial enquiry.

Therefore, the aims of this study were to:

1. examine the acceptance of parents/carers towards the use of SDF as an option for the management of caries in children's primary teeth in secondary care dental settings in the UK and the United States and
2. determine which factors may affect their acceptance of the use of SDF.

## 2 MATERIALS AND METHODS

Ethics approval was granted by an NHS research ethics committee (Ref.: 21/SW/0018) for the study in Sheffield. In Colorado, approval was granted from the Colorado Multiple Institutional Review Board (Ref.: 20‐2553).

The study participants were parents or primary caregivers of children aged 1–8 years attending the paediatric dental departments of the Charles Clifford Dental Hospital, Sheffield, UK, and the Children's Hospital Colorado, Aurora, Colorado, USA. Written consent was obtained from parents or primary caregivers. Patients and the public supported the development of study documentation including information leaflets and consent forms through providing written and verbal feedback.

### 2.1 Sample and data collection

In Sheffield, a sample size of 113 was calculated to allow the evaluation of the effects of different variables on parental/caregivers' acceptance with statistical significance set at *p* < .05 and power of 80%. The questionnaires were self‐administered; a researcher, however, was present to support the participants if this was requested. Parents/caregivers were excluded if there was no interpreter available to help with any English‐language difficulties. Data were collected from 24 March 2021 to 28 April 2021. A convenience sample was sought from parents/carers attending clinics on days when the investigator (LT) was available to recruit participants.

In Aurora, Colorado, the sample size was determined as 104. The Colorado questionnaire was available in both English and Spanish. Again, a convenience sample was included, based on parents'/carers' approach in the clinic waiting room at the dental centre by the investigator (TT/CS/ARW). Data were collected from 01 June 2021 to 31 December 2021.

For simplicity, participants shall henceforth be referred to simply as parents, rather than parents/caregivers.

### 2.2 Questionnaire

The questionnaire was developed in stages. In the first instance, the ‘*cavity treatment questionnaire*’ was developed and validated by the Colorado research team. A pilot study was conducted to test the reliability and validity of the instrument. No excessive skew or kurtosis was seen, indicating adequate distribution of variables. The questionnaire was adapted based on local demographics and language for both the Sheffield and Colorado populations. In Sheffield and Colorado, patient and public representatives were involved in adapting the questionnaire to ensure face validity for that specific population. This included advising on the language used and the number of questions, the content and the time taken to complete the questionnaire. This was through the discussion with a researcher, and piloting the questionnaire and providing written and verbal feedback. Examples of changes made in the UK questionnaire were the use of ‘slightly’ rather than ‘somewhat’, ‘injection’ rather than ‘shot’ and reducing the number of questions.

Socio‐demographic data (age and gender) were collected for both parents and their child. Questions were asked about the importance parents placed on the aesthetic consequences of dental treatment, and their perspectives of their child's treatment experience such as whether general anaesthetic (GA), local anaesthetic (LA) or the use of rotary instruments are important factors for the parents when making treatment decisions. The questionnaire included photographs of teeth treated with preformed metal crowns and SDF. Parents were asked whether they would theoretically accept SDF treatment for their child. The Sheffield questionnaire had 15 items as patient and public representatives recommended shortening the length of the questionnaire to aid completion. The Colorado questionnaire also asked questions pertaining to other treatment modalities and collected different demographic data (race and ethnicity), in addition to the above. The questionnaire contained 25 items. There were minor differences in some questions; the main question related to the acceptance of SDF, however, was the same for both the questionnaires. The SDF acceptability questions are provided in the supplemental materials. The responses were recorded using a 5‐point Likert scale (an example of the response options is as follows: strongly agree, slightly agree, neither agree nor disagree, slightly disagree and strongly disagree).

Owing to some differences in the final version of the questionnaire used in either setting, particularly relating to socio‐demographic factors, results for the two countries are presented separately. Socio‐demographic factors were measured using different methods, which were not directly comparable. Furthermore, owing to feedback when developing the Sheffield questionnaire, some questions were changed and the questionnaire was shortened; as a result, it was not possible to analyse the two data sets together. These data were analysed differently owing to the different format of the survey tools and local statistical advice.

### 2.3 Data analysis

For the Sheffield data, analysis was made through both simple descriptive and inferential statistics. The acceptability of SDF was calculated for both posterior and anterior teeth through frequencies and percentages. Logistic regression was used to determine whether the following factors affect acceptability: parent age, parent ethnicity, socio‐economic status, parent gender, child gender, child age, parent concern about GA, parent perception of how their child would cope with LA and parent's value placed on the aesthetics of their child's teeth.

Colorado data were analysed using the chi‐squared test or Fisher's exact test to compare differences between subgroups. Univariate analysis and linear multivariate model analyses were conducted to evaluate the acceptability of SDF according to independent variables. All of the independent variables with univariate association of *p* ≤ .2 with the outcome variable were included in the multivariable linear regression.^19^, ^20^

## 3 RESULTS

### 3.1 Sheffield

In total, 113 parents were recruited (response rate of 77%). Parental age range was 24–70 years (mean = 36), and children's age range was between 1 and 8 years (mean = 6 years old). The proportion of female respondents was higher (74.4%) than male (25.6%). The sex distribution of included children was more balanced, with 48.7% male and 51.3% female. Overall, 55% of the children were living in the most socio‐economically deprived areas of England. Participants came from a range of different ethnic groups as follows: 11.5% Asian; 6.2% Black; 7.8% mixed ethnicity/multi‐ethnic, 0.9% Turkish and 68.1% White. For 1.7% of respondents, ethnicity was indicated as ‘other’ but not specified, and there were missing data for 4.4%. The number of decayed, missing and filled primary teeth for children in the study ranged from 1 to 16 (mean = 7). Parents reported the previous dental attendance pattern of their child as 2.7% not previously attended; 9.7% attended only with problems; 69.9% were regular attenders; 15.9% attended sometimes; and data were missing for 1.7%.

Parents were asked whether they would accept SDF for anterior teeth or posterior teeth. The levels of agreement with these statements are shown in Table 1.

**TABLE 1:** Parental acceptance of silver diamine fluoride (SDF) treatment for anterior and posterior primary teeth (Sheffield data, *n* = 113; Colorado data, *n* = 104).

	Positive		Neutral		Negative	
Acceptance of SDF treatment						
Posterior tooth	Sheffield	Colorado	Sheffield	Colorado	Sheffield	Colorado
	73%	72%	21%	6%	6%	22%
Anterior tooth	Sheffield	Colorado	Sheffield	Colorado	Sheffield	Colorado
	58%	58%	22%	7%	20%	36%

Participants were also asked about their theoretical satisfaction with the aesthetics of SDF when used on anterior and posterior teeth. For both anterior and posterior teeth, a lower proportion of parents reported accepting discolouration following the SDF use than the overall number who stated that they would accept SDF as an intervention. This suggests that parents would potentially accept the intervention, even if they had concern about aesthetics. Parents were asked whether the way SDF looked ‘was ok with’ them, for both anterior and posterior teeth. The responses were more aligned for posterior teeth than for anterior teeth, with 73% finding the treatment acceptable; 69% indicating the appearance was acceptable; 21% and 22%, respectively, being neutral; and 6% not accepting the treatment for their child, but 9% finding the aesthetics unacceptable. For anterior teeth, there was a larger discrepancy, in which 58% stated that they would accept the treatment and 42% indicated that the aesthetics were ok with them. Those who were neutral to accepting the treatment were 22%, and neutral with aesthetics 30%, with 20% not accepting the treatment and 28% not finding the treatments satisfactory. These results indicate that although parents were not satisfied with the aesthetics of SDF, they would still potentially accept the treatment.

When examined through logistic regression, the only parameter that had a statistically significant effect on the acceptance of SDF was parents having less concern about posterior aesthetics (*p* = .013; Tables 2 and 3). Concern about the use of the rotary instruments, LA or GA, did not have an effect, and neither did the demographic details examined.

**TABLE 2:** Ordinal logistic regression to determine factors associated with parental acceptance of silver diamine fluoride for their child's anterior teeth (Sheffield data, *n* = 113).

Parameter	Estimate	Standard error	Significance
Child deprivation decile	0.121	0.075	.108
Child age	0.073	0.110	.508
Child gender			
Male	0.241	0.370	.515
Female	0.00	n/a	n/a
Parent gender			
Male	−0.63	0.483	.886
Female	0.00		
Parent ethnicity			
White	0.854	0.790	.280
Mixed	0.754	1.009	.455
Black	0;135	1.042	.897
Asian	1.126	0.918	.220
Other	0.00	n/a	n/a
Other factors			
Concern about GA			
Low or no concern	−0.290	0.431	.502
Neutral	0.024	0.622	.969
Higher concern	0.00	n/a	n/a
Concern about LA			
Low or no concern	0.430	0.865	.619
Neutral	−0.259	0.509	.611
Higher concern	0.00		
Concern about drill			
Low or no concern	−0.972	0.823	.238
Neutral	−0.545	0.468	.244
Higher concern	0	n/a	n/a
Anterior tooth aesthetic concern			
Low or no concern	0.635	0.503	.207
Neutral	0.342	0.493	.481
Higher concern	0.00		

*Note*: Test: Ordinal logistic regression, significance level *p* < .05.

Abbreviations: GA, general anaesthetic; LA, local anaesthetic.

**TABLE 3:** Ordinal logistic regression to determine factors associated with parental acceptance of silver diamine fluoride for their child's posterior teeth (Sheffield data, *n* = 113).

Parameter	Estimate	Standard error	Significance
Child deprivation decile	0.077	0.079	.332
Child age	−0.139	0.119	.244
Child gender			
Male	0.021	0.394	.957
Female	0.00	n/a	n/a
Parent gender			
Male	−0.242	0.466	.603
Female	0.00	n/a	n/a
Parent ethnicity			
White	0.933	0.834	.258
Mixed	−0.273	1.066	.867
Black	−0.035	1.083	.817
Asian	0.307	0.952	.742
Other	0.00	n/a	n/a
Other factors			
Concern about GA			
Low or no concern	0.219	0.473	.643
Neutral	−0.170	0.662	.797
Higher concern	0.00	n/a	n/a
Concern about LA			
Low or no concern	0.195	0.911	.830
Neutral	−0.710	0.545	.897
Higher concern	0.00	n/a	n/a
Concern about drill			
Low or no concern	−0.286	0.865	.741
Neutral	−0.669	0.488	.170
Higher concern	0.00	n/a	n/a
Posterior tooth aesthetic concern			
Low or no concern	1.304	0.522	.013
Neutral	−0.310	0.491	.527
Higher concern	0.00	n/a	n/a

*Note*: Test: Ordinal logistic regression, significance level *p* < .05.

Abbreviations: GA, general anaesthetic; LA, local anaesthetic.

### 3.2 Colorado

Questionnaire data were collected from 104 parents of a child seen at the dental centre at the Children's Hospital Colorado in Aurora, Colorado. The response rate was 92%, and parents were 28–70 years old (mean = 35). The vast majority of participants were female (94.3%). The proportion of different ethnicities represented within the study population was as follows: 6.7% White; 10.6% Black; 66% Hispanic; and 16.4% indicated as ‘other’. The parental‐reported attendance pattern of their child was that 6.7% received care only for concerns, that 82.7% were regular attenders and that 10.6% attended sometimes.

Responses from the Colorado participants showed that 58% would theoretically accept SDF treatment on their child's anterior teeth and that 72% would accept SDF treatment on the posterior teeth (Table 2). Concerns about aesthetics decreased the SDF acceptance overall (*p* = .0065) in anterior (*p* = .023) and posterior teeth (*p* = .108; Table 1).

Tables 4 and 5 show the results for the factors associated with parental acceptance of SDF on anterior and posterior teeth. Factors that had an impact included a history of pain, which increased the acceptance of SDF (*E* = −1.52; *p* = .022). Regarding increase in concern about the anterior teeth aesthetics (*E* = −0.88; *p* = .0080) and concern about rotary instrument use (*E* = 0.52; *p* = .032), SDF acceptance for the treatment of anterior teeth decreased.

**TABLE 4:** Multivariate regression to determine factors associated with parental acceptance of silver diamine fluoride for their child's anterior teeth (Colorado data, *n* = 104).

Parameter	Estimate	Standard error	Significance
Parent gender			
Female	−5.64	2.30	.0160
Male	0.00	n/a	n/a
Parent race/ethnicity			
White	−1.04	2.18	.6353
Black	−2.48	1.89	.1929
Hispanic	−2.19	1.37	.1143
Other factors			
Concern about local anaesthesia	0.45	0.45	.3187
Concern about dental drill	−0.55	0.42	.0329
Anterior teeth aesthetics concern	−0.85	0.34	.0080
History of pain	0.83	0.95	.3879
Time spent away from work	−0.62	0.33	.0603

*Note*: Linear multivariable modelling *p* < .0001 for the overall the model.

**TABLE 5:** Multivariate regression to determine factors associated with parental acceptance of silver diamine fluoride for their child's posterior teeth (Colorado data, *n* = 104).

Parameter	Estimate	Standard error	Significance
Parent gender			
Female	−4.19	2.18	.0586
Male	0.00	n/a	n/a
Parent race/ethnicity			
White	−0.82	2.10	.6978
Black	−1.37	1.82	.4548
Hispanic	−0.54	1.32	.6825
Other factors			
Concern about local anaesthesia	0.40	0.43	.3526
Concern about dental drill	0.32	0.40	.4282
Posterior teeth aesthetics concern	−0.44	0.35	.2081
History of pain	−1.51	0.65	.0227
Time spent away from work	−0.28	0.31	.3806

*Note*: Linear multivariable modelling *p* < .0001 for the overall the model.

## 4 DISCUSSION

The first finding of significance is the similarity in the reported rates of acceptance of SDF between the two populations. Although it was not possible to statistically compare the two populations, this is nonetheless an interesting finding, contrasting with the previously published disparities in acceptance rates from the research conducted in different countries.^17^ Interestingly, of the factors assessed, parental ethnicity, age, gender, socio‐economic status and concern about the use of rotary instruments, GA or LA, did not affect parental acceptance in the UK, nor did parental ethnicity, concern about LA, history of pain or time away from work affect parents' acceptability in the United States. This was surprising given one of the benefits of SDF often cited is that it is minimally invasive, not requiring the use of LA, whereas parents' concern about this did not significantly affect SDF acceptance. The findings from this questionnaire study are broadly similar to those found in a US study by Crystal et al.,^18^ in which the overall parental acceptance rates for SDF application were 67% for posterior primary teeth. Data from the present study, however, revealed higher acceptance rates for SDF treatment of anterior teeth (58%) than those reported by Crystal et al. (29.7%).^18^ Furthermore, in terms of factors that affected acceptability described by Crystal et al.,^18^ the need for pharmacological behaviour management affected the acceptability of SDF in the US sample, whereas in the UK sample, greater parental concern about the use of GA did not significantly impact SDF acceptance. Owing to the use of different survey instruments, it is difficult to undertake direct comparisons between the findings of the present study and those of other studies.

Nonetheless, the results are promising in that SDF was generally acceptable to the majority of parents of young children questioned. Furthermore, our study found that some parents reported that they would accept SDF for their child, despite not finding the aesthetics satisfactory. Qualitative data from Kyoon‐Achan et al. support the finding that parents may accept this treatment option despite dissatisfaction with the aesthetics.^21^ This demonstrates the importance of discussing SDF as a potential treatment option where indicated, highlighting both its risks and benefits to parents and children.

There is a paucity of data regarding the acceptability of SDF in the UK and indeed Europe, and this study provides evidence to address this research gap. There is also a lack of data from the Western region in the United States. In both cohorts, we considered how demographic details along with attitudes to different elements of treatments affected the acceptance of SDF. To provide additional information, we asked about preferences related to aesthetics, local anaesthesia and the use of air rotary instruments to form a more complete picture of factors, which may be related to the acceptance of SDF.

A strength of this study was the involvement of patient and public representatives in the design of the study information and the questionnaire. This improved the quality of the questionnaire to ensure that the questions were clear and relevant to their target audience. The questionnaires were well‐completed, and it is speculated that involving parents in their design may have contributed to the high quality of data collected. Furthermore, the patient's support in producing study documentation may have improved the response rate by ensuring study information was clear. The response rate was 77% in Sheffield and 92% in Colorado, which compares favourably with previous questionnaire‐based studies in similar populations (61.2% in a similar UK cohort).^22^

In terms of study limitations, it has to be acknowledged that findings from the two settings were not directly comparable because of some modifications to the questionnaire used, and the fact that they were issued at different times. The Sheffield questionnaire was shorter based on the advice of public and patient representatives; as such, the parameters that may have affected acceptance were prioritised over direct comparisons with other treatments. Furthermore, the respondents were parents of children who had attended the department for a range of treatments, including assessments and other treatment modalities. As such, the majority are unlikely to have had first‐hand experience of the treatment or the resultant aesthetics. Their responses were hypothetical and based on photographs and descriptions of the proposed treatments. Nevertheless, if participants had only been drawn from those whose child had received SDF, this would have led to positive bias as only those who had accepted the treatment would have been included. Notwithstanding, the majority of parents had children with early childhood caries and were from deprived groups most at risk of caries, making them a likely target population for future SDF treatment. Patients attending these clinics in the UK are those who have been referred for treatment and by virtue of requiring referral and are generally a higher need group who have not managed treatment in a primary care setting, and as a result, they differ from the general population. For those attending in the US clinics, the majority of paediatric patients are from underserved groups with higher oral disease and treatment needs compared with the overall population.

Data were collected over a 5‐week period in the UK, and 6 months in the United States. The difference was due, in part, to researcher availability. In the UK, the researcher collecting data had more time per week in order to collect data (equivalent to 1 day), whereas this was 2 days per month for the US researcher, facilitating quicker data collection in Sheffield. As such, despite the lower response rate, data collection was completed over a shorter time period in the UK.

This work provides valuable evidence to clinicians who hold the belief that SDF is unacceptable to families because of consequent compromised dental aesthetics.^13^, ^14^, ^15^, ^16^ The positive aspects of SDF, however, may outweigh this limitation, particularly in terms of the reduced demands on young children who may lack the level of cooperation needed for more traditional techniques. The potential of SDF to avert caries progression, pain and infection and ultimately the need for invasive procedures using pharmacological interventions are undisputed benefits. Future international qualitative research with young children and their parents, along with dental professionals, would add further evidence regarding the acceptability of SDF as a caries management strategy for young children.

The use of SDF was found to be acceptable to the majority of parents in the two study populations. An additional finding from the United States was that SDF appeared to be more acceptable than traditional caries management techniques. Parental attitudes towards aesthetics understandably may impact the rates of acceptance, although it is important to note that based on the results of our study, parents who are displeased with the resultant staining of SDF may still opt to choose this treatment for their child. Parent's preference for less invasive techniques may outweigh the aesthetic concerns. Additionally, parents may have fewer concerns about aesthetics as carious involvement also imparts a less aesthetic appearance to involved teeth.

## AUTHOR CONTRIBUTIONS

LT was involved in the study design, data collection, analysis and preparation of the manuscript. SC was involved in the data collection. ZM, HR, ARW and TT were invovlved in the study design, data analysis, and preapration of manuscript.

## FUNDING INFORMATION

Funding was received through the British Society of Paediatric Dentistry and the Faculty of Dental Surgery Royal College of Surgeons of England.

## CONFLICT OF INTEREST STATEMENT

The authors declare no conflicts of interest. The study received ethics approval in both studies. Parents provided written consent to take part in the study.

## Supporting information


Appendix S1.

## Data Availability

Data are available upon request.
