# Supplementary material for: Parental acceptability of silver diamine fluoride: The UK and US experiences
Source: Int J Paediatr Dent. 2024 Apr 26;35(1):13–21. doi: 10.1111/ipd.13195 (PMC11626544; doi:10.1111/ipd.13195)
Supplement: Supplementary file 1 — Appendix S1. [file IPD-35-13-s001.docx]

**
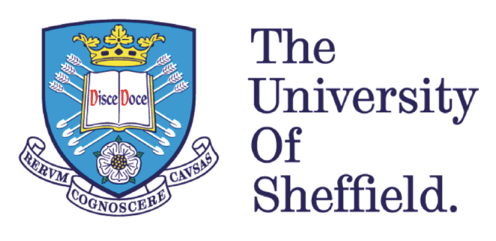

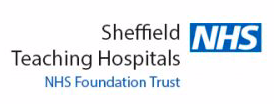
**

**Welcome to the Dental Decay Treatment Questionnaire**

STH Project Number: 21328

**Instructions for the Dental Decay Treatment Questionnaire**

Thank you for taking part in our study.

As you answer the questions, please keep

in mind:

• When a question asks about "your child," please answer for only one of your children.

• Answer all questions keeping in mind the same child.

• All questions are about baby teeth.

For each question, please choose the answer that is most like how you feel, think, or act ‐‐

even if it isn’t **exactly** like you.

Then put a cross in the box next to it like this.

Only cross **ONE** box for each question.

Researcher to complete participant ID:

| Participant ID |  |  |  |  |  |  |
| --- | --- | --- | --- | --- | --- | --- |

Please complete the questions below.

If you have any questions please ask.

**First, please answer some questions about you.**

What is your age in years?

Enter age

Are you?

Female

Male

Other

Prefer Not to Say

What is your ethnicity?

White

Mixed/multi-ethnic

Black

Asian

Other

**Now please answer these questions about your child.**

**Does your child have dental decay (holes in their teeth)?**

Unsure

Yes

No

**Do they go to see the dentist?**

Never

Only when there are problems

Sometimes

Regularly

**Have they had dental treatment (eg fillings/tooth removal) before?**

Unsure

Yes

No

The pictures below show what tooth decay looks like in baby teeth.

Decay can cause holes in the teeth or make them look brown or black.

If left without treatment, it can lead to children getting pain or infection.


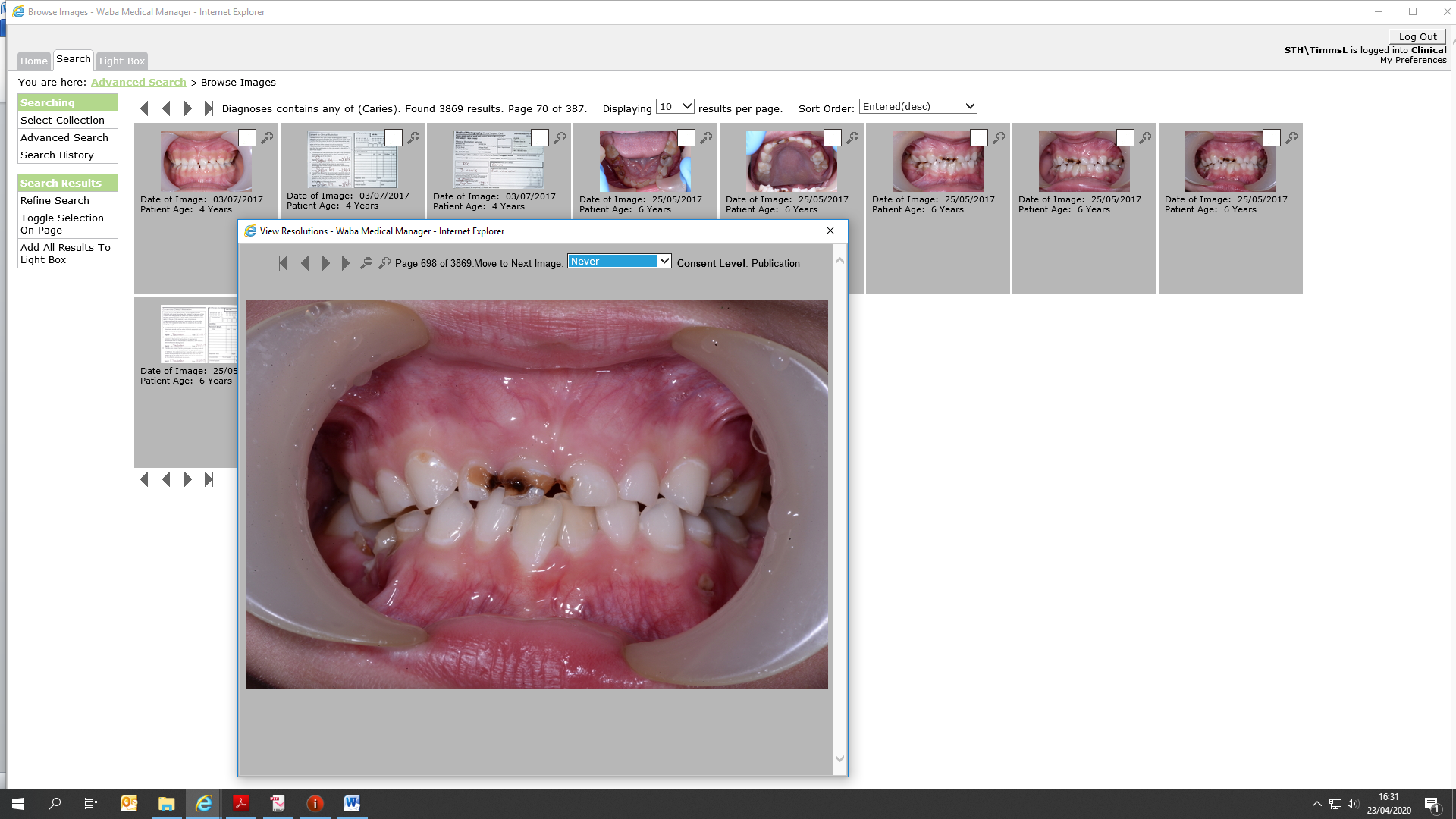


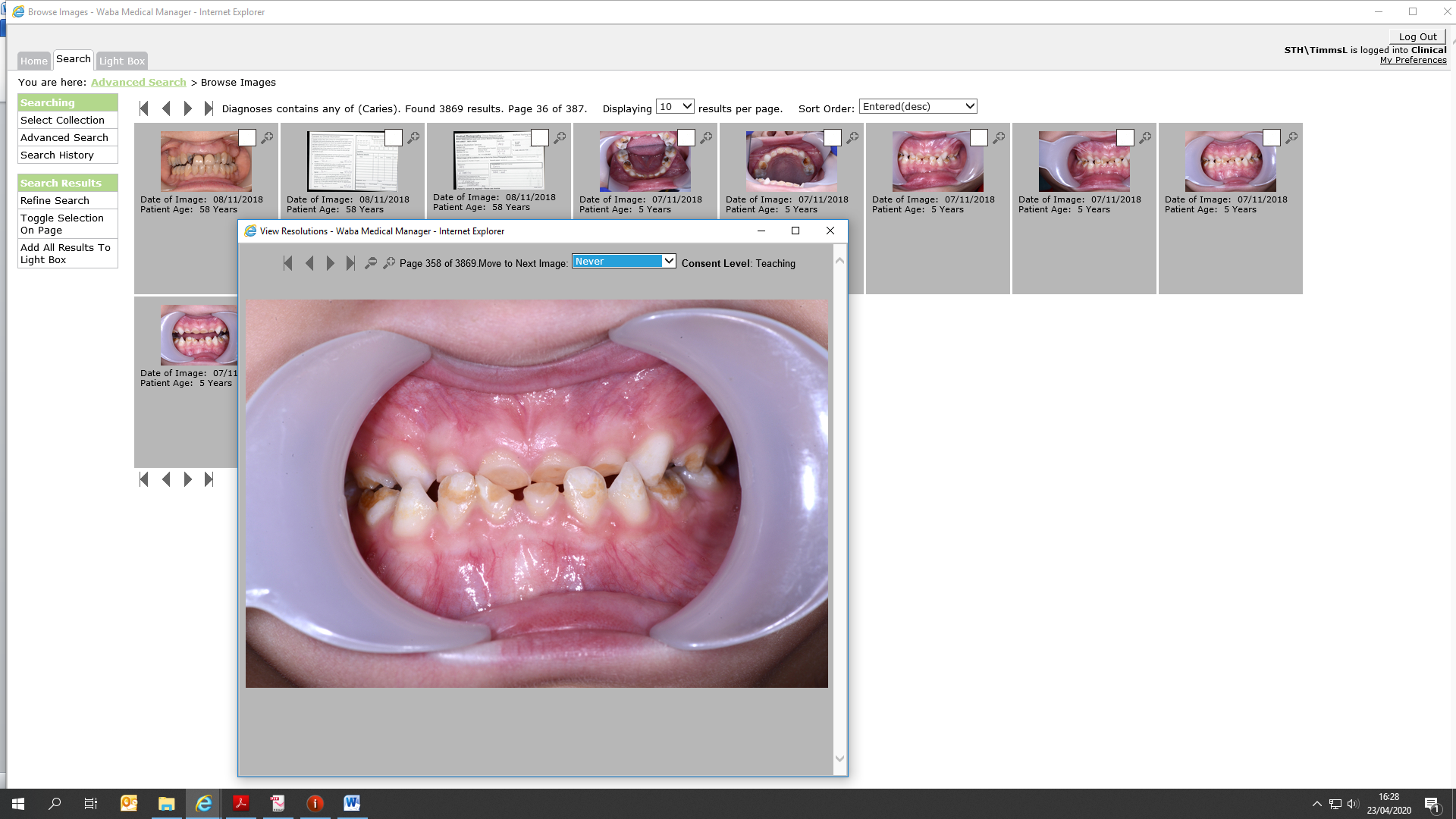

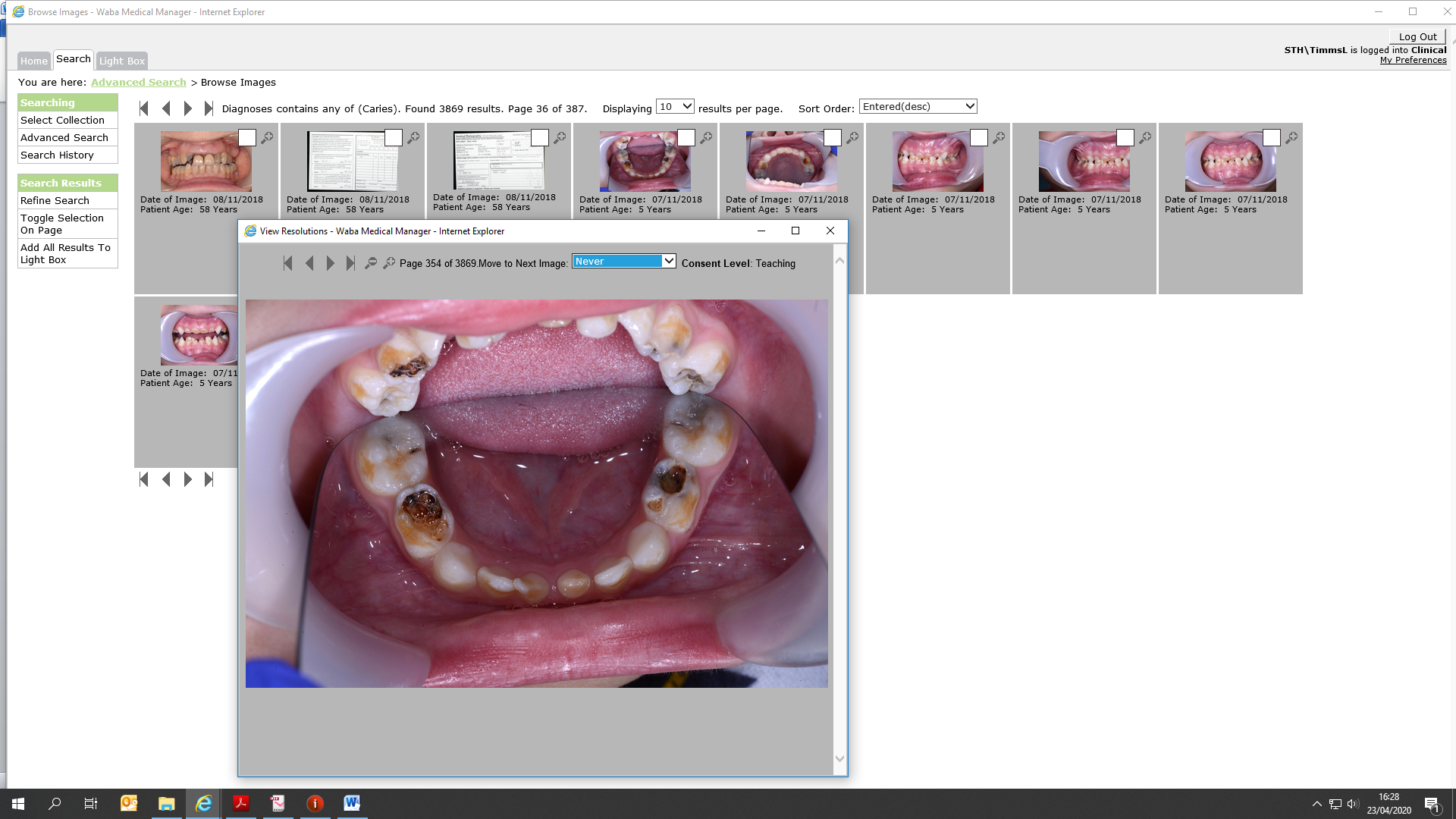


**The next questions are about what you think is important about treating your child’s tooth decay**

**Sometimes children need a general anaesthetic, this will make your child fall asleep and remain asleep during the treatment (they will be unaware of what is happening).**

**Are you concerned about this?**

Not at all concerned

Not very concerned

Neutral

A little concerned

Very concerned

**Sometimes children need to have an injection (local anaesthetic – your child will stay awake) for the decay to be treated. Would this be a problem for your child if they needed an injection?**

Strongly Agree

Slightly Agree

Neither Agree nor Disagree

Slightly Disagree

Strongly Disagree

**The dentist needs to use a very small drill for some treatments. Would this be a problem for your child?**

Strongly Agree

Slightly Agree

Neither Agree nor Disagree

Slightly Disagree

Strongly Disagree

**Some treatments make front teeth look darker, some fill the tooth with white material, and some leave a gap. Are you concerned about how your child’s front teeth will look after treatment?**

Not at all concerned

Not very concerned

Neutral

A little concerned

Very concerned

These teeth are back baby teeth


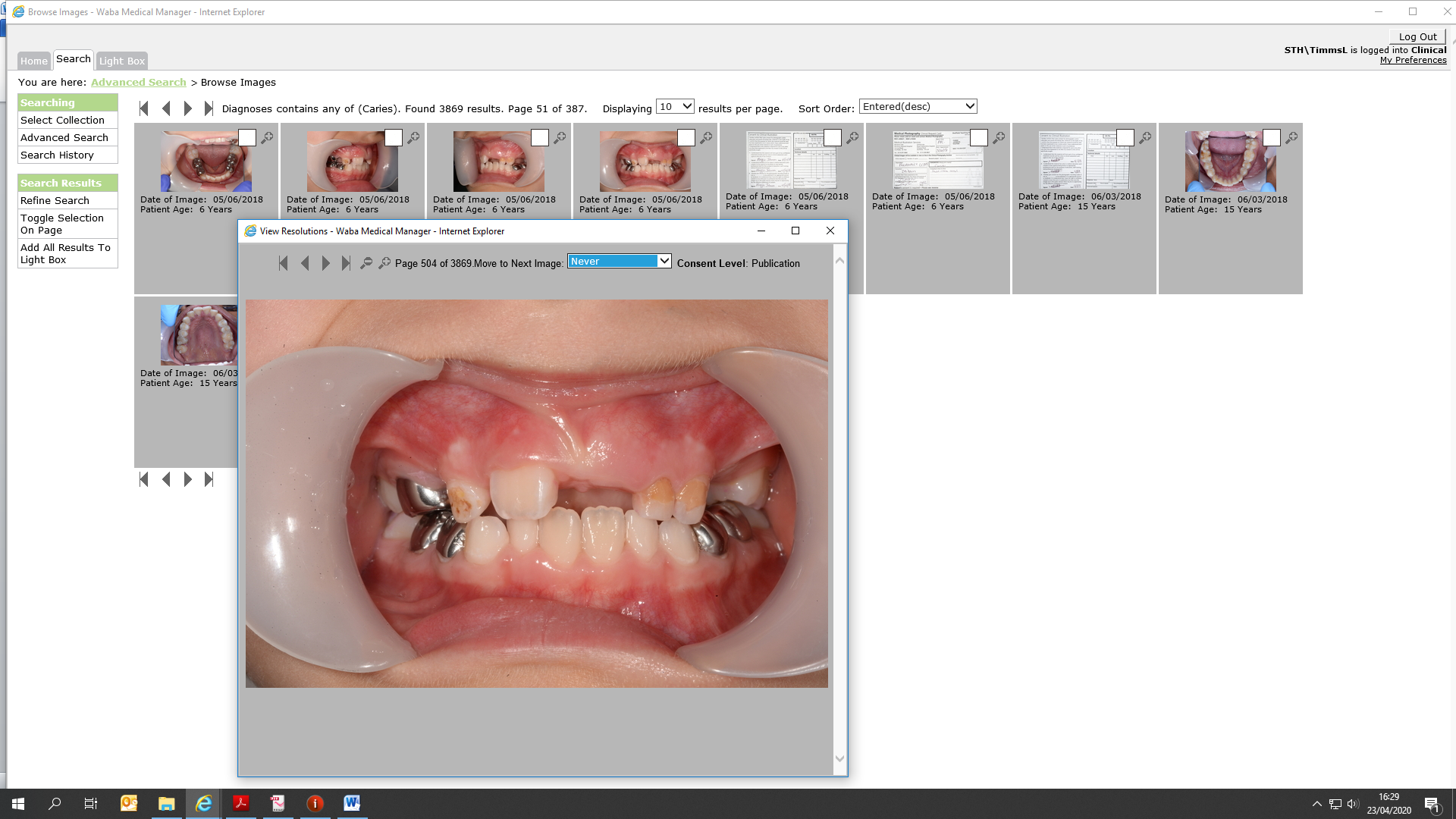


**Some treatments make back teeth look darker, some cover the teeth with metal, and some leave a gap. Are you concerned about how your child’s back teeth look after treatment?**

Not at all concerned

Not very concerned

Neutral

A little concerned

Very concerned

**Silver diamine fluoride (SDF) Liquid Treatment**

SDF is a liquid painted onto teeth.

It does not require local anaesthetic (injection), general anaesthetic or drilling of the tooth. The treatment takes 5 minutes in the dental chair.

The SDF stains the decayed area of the tooth black, as shown in the photographs below. It stops the decay getting worse. It may need to be reapplied once or twice a year.

SDF on front teeth:


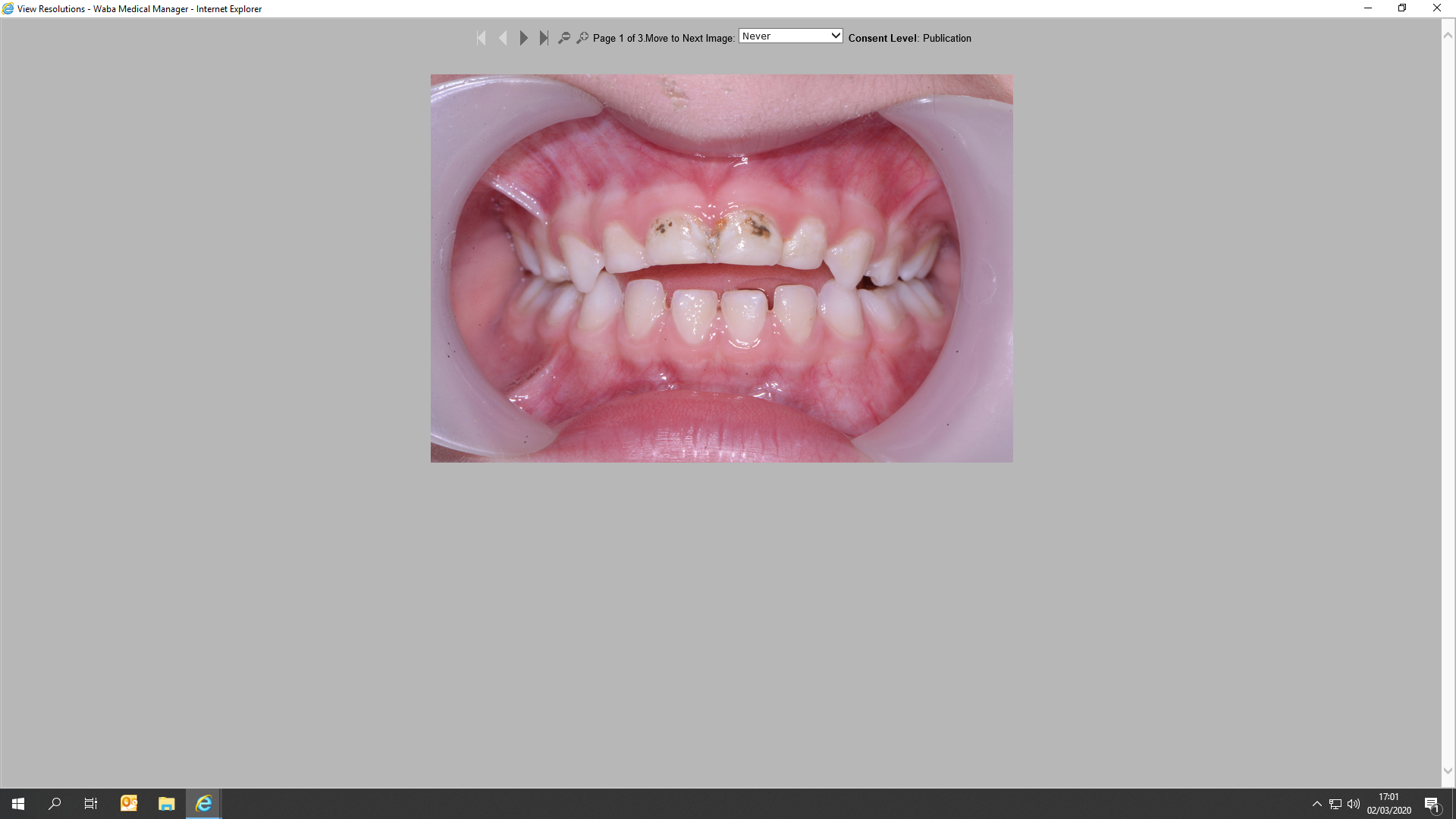


SDF on back teeth:


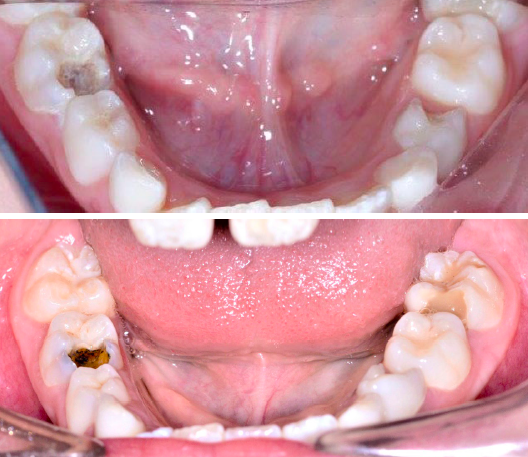


**I would accept SDF liquid treatment if my child has a hole in a front tooth**

Strongly Agree

Slightly Agree

Neither Agree nor Disagree

Slightly Disagree

Strongly Disagree

**The way SDF liquid treatment looks in a front teeth is OK with me**

Strongly Agree

Slightly Agree

Neither Agree nor Disagree

Slightly Disagree

Strongly Disagree

**I would accept SDF liquid treatment if my child has decay in a back tooth**

Strongly Agree

Slightly Agree

Neither Agree nor Disagree

Slightly Disagree

Strongly Disagree

**The way SDF liquid treatment looks in a back tooth is OK with me**

Strongly Agree

Slightly Agree

Neither Agree nor Disagree

Slightly Disagree

Strongly Disagree

**Thank you for completing the questionnaire.**

**Please let a staff member know you have finished.**
